# Chikungunya vaccine approval in Brazil: navigating through epidemiological challenges and immunisation strategies

**DOI:** 10.1590/0074-02760260088

**Published:** 2026-07-31

**Authors:** Claudia Renata dos Santos Barros, Sandra Coccuzzo Sampaio, Maria Carolina Elias, Svetoslav Nanev Slavov

**Affiliations:** 1 Instituto Butantan, Centro de Vigilância Viral e Avaliação Sorológica, São Paulo, SP, Brasil

**Keywords:** Chikungunya virus, arboviruses, vaccine, spatial epidemiology, health inequities, Brazil

## Abstract

Chikungunya virus (CHIKV) can cause acute arboviral illness, usually accompanied by severe polyarthralgia. In 2025, live-attenuated CHIKV vaccine Ixchiq^®^ (Valneva, Saint-Herblain, France) was authorised for use in Brazil, where CHIKV often co-circulates with other arboviruses. A central public health question emerges: how should this vaccine be optimally deployed in a country characterised by hyperendemic transmission, frequent underdiagnosis, and marked regional disparities? We conducted an epidemiological analysis using notified CHIKV cases from the Notifiable Diseases Information System (SINAN) and socioeconomic indicators from the Brazilian Human Development Atlas. This framework enabled the identification of municipal clusters with shared epidemiological and socioeconomic profiles, allowing exploration of the relationship between disease notification rate and social health determinants. Our results showed that CHIKV transmission exhibited a cyclic pattern with geographic expansion toward the Southeast/South — Brazil’s most densely populated regions characterised by low population immunity. Cluster analyses showed that the greatest disease burden is concentrated in socioeconomically disadvantaged municipalities, particularly in the Northeast, where healthcare disparities may further impact diagnostic capacity. Based on the obtained results, we believe in a hybrid immunisation strategy starting from high-incidence municipalities in the northeast and reaching major urban centres in the southeast in order to prevent larger outbreaks. Implementation must account also for current vaccine contraindications in high-risk groups and be guided by real-time epidemiological and entomological surveillance.

Chikungunya virus (CHIKV) can cause an acute febrile disease with symptoms including high fever, myalgia, headache, photophobia and rash. A prominent complication of CHIKV infection is polyarthralgia often leading to disabling complications especially involving the large joints.^1^ ^,^ ^2^ The etiological agent, CHIKV belongs to the genus *Alphavirus*, *Togaviridae* family and is transmitted by the bite of mosquitoes belonging to the *Aedes* genus, especially *Ae. aegypti* e *Ae. albopictus*.^3^

CHIKV was initially restricted to limited outbreaks in Africa and Asia. However, in 2005, large-scale epidemics occurred across vast geographic regions of the Indian Ocean, highlighting the potential of this virus for global expansion.^4^ In 2013, CHIKV was introduced into the Americas. The first reported cases in Brazil occurred in 2014, following two independent introductions of the virus into different states. The Asian genotype was introduced in the state of Amapá, while the East/Central/South African (ECSA) genotype was introduced in the State of Bahia.^5^ Initially, CHIKV transmission was primarily concentrated in northeast Brazil, where the ECSA genotype had been introduced. However, in 2019, CHIKV started a dissemination towards southeast Brazil, where a growing number of cases was recorded. Notably, this region has the highest population density in the country and includes Brazil’s largest cities.^6^

No specific CHIKV treatment exists. A significant advancement in the CHIKV prevention, particularly regarding severe disease and complications, occurred with the development and regulatory authorisation of the live-attenuated vaccine IXCHIQ^®^ in several countries and regions including the European Union, United Kingdom, Canada, and Brazil.^7^ In Brazil, IXCHIQ was approved in 2025 by the Brazilian Health Regulatory Agency (ANVISA) and will be manufactured by the Butantan Institute, São Paulo (https://saude.sp.gov.br/coordenadoria-de-controle-de-doencas/noticias/). This marks the first attempt for implementation of a CHIKV vaccine in a hyperendemic country, representing a historic milestone for the control of this infection in low- and middle-income settings. Nevertheless, the introduction of the IXCHIQ vaccine in Brazil may pose different challenges including equitable access across the country’s diverse regions and populations.

Therefore, we performed an epidemiological investigation which outlines the main trends in CHIKV dissemination, notification rate and the identification of at-risk populations in Brazil. These factors are crucial for conforming a possible national immunisation strategy and addressing to the associated public health challenges.

## Notification rates of CHIKV in Brazil

A retrospective study was conducted using secondary data on notified, suspected and confirmed CHIKV cases reported between 2016-2023. Epidemiological data were obtained from the Brazilian National Notifiable Diseases Surveillance System (SINAN) through the Department of Informatics of the Unified Health System (DATASUS). In this study, notification rates are used as a proxy for disease burden, acknowledging that these figures may not reflect true incidence due to inherent underreporting and regional variations in surveillance systems. The study period was chosen based on the public availability of data starting in 2016, long after the introduction of this virus in Brazil. This time frame additionally allowed us to evaluate the potential changes in reporting patterns associated with the Coronavirus disease 19 (COVID-19) pandemic.^8^ Demographic and socioeconomic information at municipal level was retrieved from the Brazilian Institute of Geography and Statistics (IBGE), based on the 2010 national census, given that complete data from the 2022 census were not yet available at the time of analysis. The following variables were considered: (i) annual estimated population (2016-2023); (ii) municipal human development index (MHDI); (iii) MHDI - income component; (iv) MHDI - education component; (v) MHDI - longevity component.

The database was processed and refined before analysis through procedures including standardisation, consistency checks, duplicate removal, and the handling of missing values. Outliers in case counts and notification rates were evaluated using visual inspection and descriptive statistical analyses.

The normality of the distribution of notified cases was tested using the Shapiro-Wilk test. The notification rate was calculated for the 5,570 Brazilian municipalities with valid notifications recorded for all years within the study period. The used formula was (proxy notification rate):

Notification rates were reported for the entire country as well as for each of the five major geographic regions of Brazil (Central-West, North-East, North, South-East, and South).

## Cluster analysis

Following descriptive analysis, a cluster analysis was performed to identify patterns of similarity among municipalities based on CHIKV notification rates. A non-hierarchical k-means clustering method was applied, and the optimal number of clusters was determined using the elbow method. After determining the cluster number, a descriptive characterisation of each group was conducted using the following variables: notification rate, MHDI, and its components — income (MHDI-income), education (MHDI-education), and longevity (MHDI-longevity) — to interpret the socio-epidemiological profiles of the resulting clusters.

All analyses were conducted using the RStudio statistical environment, employing the dplyr, ggplot2, and factoextra packages.

## Ethics committee statement

This study was based on publicly available, anonymised and aggregated data obtained from official databases. No individual-level identifiable information was accessed or analysed. Therefore, according to ethical regulations, ethics committee approval and informed consent was not required.

## Notification rates of CHIKV in Brazil

**Fig. 1:**
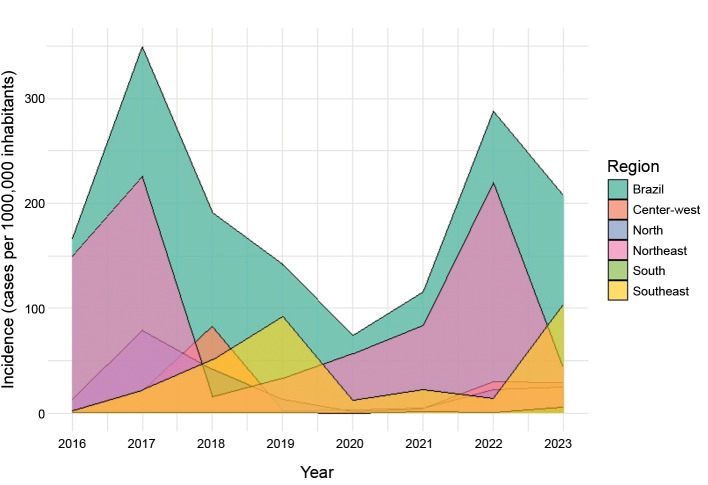
temporal trends in Chikungunya notification in Brazil and its macro-regions from 2016 to 2023.

In Fig. 1, can be observed an oscillating CHIKV epidemic pattern, with pronounced peaks occurring in alternating years, which was particularly evident in the Northeast and Southeast Brazilian regions. The Northeast region exhibited the highest notification peaks in 2016, 2017, and 2022, with rates exceeding 200 cases per 100,000 inhabitants.

The Southeast region, in contrast, showed a progressive increase in notification rate over the study period, culminating in a large peak in 2023, when it reached 100 cases per 100,000 inhabitants concentrated in the Minas Gerais State (Fig. 1).^9^

The South region, which historically reported the lowest notification rates, has also recorded higher values in recent years, although still lower compared to other regions (Fig. 1). The other Brazilian regions — North and Central-West — exhibited more limited fluctuations, with a notable increase in the Central-West in 2018. However, compared to the Northeast and Southeast regions, the latter were characterised by a more stable pattern (Fig. 1).

The aggregated national trend line highlights these epidemic cycles, with peaks in 2016, 2018, and 2022, describing the cyclic nature of CHIKV outbreaks. The shifting transmission axis towards the southeast and more recently south Brazil may serve as an epidemiological alert, indicating the urgent need to reinforce prevention and response strategies in regions previously considered at low-risk (Fig. 1).

## Cluster analysis

In Table are represented the average profiles of the three municipal clusters generated through cluster analysis based on CHIKV notification rate and the components of the MHDI: income, education, and longevity. The segmentation resulted in three groups with distinct patterns of socioeconomic and epidemiological vulnerability.

Cluster 2, which includes the majority of municipalities (n = 802), exhibits the highest average notification rate CHIKV rate (374.72 cases per 100,000 inhabitants), combined with the lowest socioeconomic indicators, including an average MHDI of 0.58 and the lowest scores for income (0.56) and longevity (0.47). This group reflects municipalities with the highest disease burden and structural vulnerability, highlighting areas that require increased public policy attention and strengthened health surveillance systems (Table).

**TABLE:** Average profiles of municipal clusters based on Chikungunya virus (CHIKV) notification rates and components of the municipal human development index (MHDI) — income, education, and longevity — for the period 2016 to 2023, Brazil, 2025

Cluster	n	Notification	MHDI	MHDI-income	MHDI-education	MHDI-longevity
1	372	123.67	0.75	0.74	0.84	0.68
2	802	374.72	0.58	0.56	0.75	0.47
3	482	238.61	0.67	0.65	0.81	0.57

In contrast, Cluster 1 represents municipalities with the lowest average notification rate (123.67 cases per 100,000 inhabitants) and the most favourable human development conditions, with an average MHDI of 0.75 and high values in the education (0.84) and income (0.74) dimensions. These municipalities demonstrate a profile of lower epidemiological vulnerability, potentially associated with better urban infrastructure, broader access to health services, and improved sanitation (Table).

Cluster 3 represents an intermediate group (n = 482), with an average notification rate of 238.61 cases per 100,000 inhabitants and an MHDI of 0.67. Its indicators reflect median socioeconomic conditions and a moderate epidemiological risk. This group may represent areas undergoing epidemiological transitions or internal heterogeneity, where more and less developed regions coexist in terms of social and sanitary structure (Table).

**Fig. 2:**
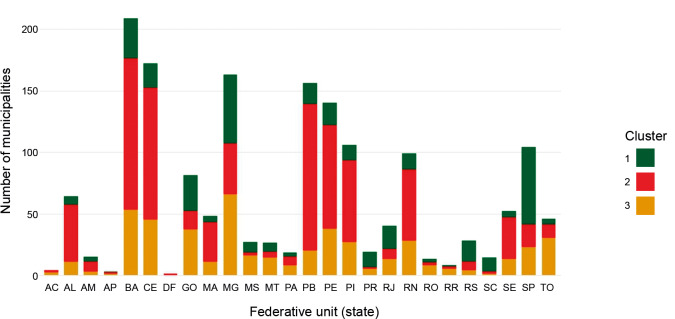
distribution of municipalities by state according to the three clusters identified through cluster analysis based on Chikungunya notification and components of the municipal human development index (MHDI), for the period 2016 to 2023. Brazil, 2025.

In Fig. 2 we represent the distribution of municipalities by state according to the three clusters identified. Notably, states such as Bahia, Maranhão, Pará, Pernambuco, and Paraíba are predominantly represented in Cluster 2, indicating a high concentration of municipalities with elevated CHIKV notification rates associated with low human development indicators.

Conversely, states like Minas Gerais, São Paulo, and Espírito Santo have positioned more municipalities in Cluster 1, reflecting more favourable socioeconomic conditions and lower epidemiological risk. This pattern may indicate a higher local health system response capacity, as well as better structural indicators. The significant presence of Cluster 3 in states such as Bahia, Goiás, Maranhão, and Mato Grosso suggests local transitions, with municipalities in intermediate conditions that require context-specific interventions (Fig. 2).

Such type of cluster distribution highlights the need for regional strategies, with an emphasis on the most vulnerable municipalities (Fig. 2), while also addressing those in transitional contexts (Cluster 3), which may face increased transmission risk if timely public health actions are not implemented.

Currently, Brazil is a hyperendemic country for CHIKV,^10^ which underscores the urgent need for coordinated control measures against this infection. In this epidemiological study, we provide investigations on the recurrence of the CHIKV outbreaks in Brazil mainly focusing on the necessity and availability of vaccination and the socio-economic drivers (clusters) that might have led to increased notification rates. Crucially, our analysis accounts for the fact that CHIKV is significantly underdiagnosed in Brazil, with diagnostic challenges and health assistance disparities varying greatly across regions. We acknowledge that notification rates serve as a proxy for disease burden rather than true infection incidence, as reporting efficiency is inherently influenced by regional variations in surveillance capacity. Given the rapid CHIKV expansion from the Northeast to the Southeast/South regions, the approval of the CHIKV vaccine by the ANVISA in 2025 is expected to gain incorporation in the Brazilian Public Health Services. Key questions remain to be discussed in regards of the vaccine application in Brazil, identifying the most critical areas for immediate vaccine applications, which populations have higher priority, and the limitations for the nationwide CHIKV program.

We observed that CHIKV outbreaks exhibit an oscillating pattern over time with two major national peaks associated with the Northeast region. Although this region remains the most affected one, a concerning trend emerged in 2023, with a shift in CHIKV outbreaks toward southeast Brazil showing intensified transmission, reaching a notification rate of 395 cases per 100,000 inhabitants.^6^ In southernmost Brazil, CHIKV transmission has occurred only sporadically, although an isolated outbreak was recorded in 2021.^11^

It is essential to distinguish CHIKV dynamics from other arboviruses like Dengue virus (DENV). Unlike the multi-serotype complexity of DENV, CHIKV transmission is fundamentally constrained by long-term immunity to a single infecting serotype. Furthermore, CHIKV has specific thermal thresholds and transmission models that do not necessarily mirror the patterns observed in the 2024 DENV epidemic, explaining its unique regional recurrence patterns with implications for economic stability, healthcare system, and challenges for vector control, underscoring the importance of adaptive public health policies, particularly in urban environments.^12^

In our study, we conducted a cluster analysis to evaluate how socioeconomic factors are associated with the CHIKV risk. This approach enabled the identification of clusters of municipalities sharing similar epidemiological and social characteristics, revealing that higher notification rates are concentrated in areas with poorer socioeconomic indicators, including low income and reduced life expectancy. We acknowledge that municipality-level data can mask intra-municipal heterogeneities of arbovirus circulation and this can simplify the complex arbovirus epidemiology. However, our findings corroborate with previous studies^13^ and also emphasise that the municipality is the primary administrative and operational unit in Brazil for public health management and implementation of vaccination strategies.

By delineating territorial patterns of vulnerability at the primary administrative level used by the Brazilian Unified Health System (Sistema Único de Saúde — SUS), this analytical technique guides the more effective allocation of resources, the targeting of epidemiological surveillance actions, and the formulation of focused public policies, especially in historically neglected regions.^14^ ^,^ ^15^ While our study focuses exclusively on standardised socioeconomic indicators, we acknowledge that the absence of association of environmental and entomological variables, such as vector density and water scarcity can be regarded as a limitation of our analysis. These factors, however, are important for investigations of the transmission dynamics of CHIKV and their inclusion is beyond the scope of this study which is focused on nationally available and standardised indicators.

A critical question is the vaccine application strategy in Brazil, particularly in light of CHIKV geographic expansion and the complex clinical landscape characterised by the co-circulation of multiple arboviruses. In many Brazilian regions, CHIKV circulation overlaps with other important arboviruses like DENV, Zika virus (ZIKV) and Oropouche. Since these infections often present with overlapping acute symptomatology, relying solely on clinical triage creates an epidemiological challenge for accurate disease tracking. This complex scenario underscores the strategic importance of a targeted CHIKV vaccine in Brazil. By preventing this specific infection, targeted vaccination would drastically reduce the overall disease burden and mitigate the long-term public health impact of CHIKV, particularly its characteristic chronic arthralgia, regardless of acute-phase diagnostic limitations.

Sustained transmission, recurrent CHIKV outbreaks^10^ and the highest prevalence rates^16^ ^,^ ^17^ in northeast Brazil are consistent with incomplete herd immunity and a potentially high vulnerability among paediatric populations. On the other hand, CHIKV spread recently to southeast Brazil, where herd immunity also remains low.^18^ In this context, we propose a hybrid immunisation strategy as a conceptual framework for prioritisation under potential resource constraints, rather than an exclusive policy recommendation. This approach is rooted in the principle of distributive justice: providing interventions where the risk of disease is objectively higher, reducing the individual disease burden and the potential for chronic sequelae in those already disproportionately affected, rather than serving as a utilitarian barrier to protect other regions. Such an approach could interrupt ongoing transmission cycles in chronically affected areas and provide protection to highly vulnerable populations, and alleviate the strain on local healthcare systems already struggling with the simultaneous management of multiple arboviral outbreaks.

The expansion of this vaccination program should be informed by surveillance data, including notification rates, seroprevalence studies, mosquito vector abundance, and population mobility patterns from endemic to non-endemic areas. Furthermore, the current clinical limitations of Ixchiq^®^ must be addressed; it is a live-attenuated vaccine and its reactogenicity profile requires careful assessment, particularly in paediatric patients, immunosuppressed individuals and older populations who may face a higher frequency of systemic adverse events. Currently, this vaccine, it is not indicated for certain high-risk groups, such as neonates, pregnant women, and immunocompromised individuals, due to the present lack of comprehensive safety and efficacy data for these specific populations. This restriction poses a significant challenge, as these groups, along with the elderly, may be more susceptible to vaccine-related adverse events and are often the most susceptible to severe CHIKV complications.

The SUS and the National Immunisation Program (Programa Nacional de Imunizações — PNI) represent two of the most comprehensive public health frameworks globally, offering a robust foundation for the equitable deployment of vaccines. The ethical imperative of CHIKV immunisation in Brazil lies in integrating epidemiological evidence with principles of social justice, while navigating the shifting paradigm of public vaccine acceptance. As individual risk-benefit perceptions increasingly outweigh traditional collective benefits, addressing this shift requires radical transparency in risk communication. By identifying high-burden ‘hotspots’, our framework allows health authorities to provide precise, community-level data, enabling individuals to make informed decisions by weighing their localised infection risk against the vaccine’s safety profile. However, the feasibility and effectiveness of a potential mass CHIKV vaccination campaign will critically depend on the reinforcement of surveillance systems across all geographic regions. This is particularly vital for monitoring real-world safety profiles and adverse events, enabling the dynamic adaptation of immunisation strategies in response to evolving epidemiological conditions and emerging clinical evidence regarding under-represented populations in initial trials.

## Data Availability

The contents underlying the research text are included in the manuscript.
