# Supplementary material for: Chikungunya vaccine approval in Brazil: navigating through epidemiological challenges and immunisation strategies
Source: Mem Inst Oswaldo Cruz. 2026 Jul 31;121:e260088. doi: 10.1590/0074-02760260088 (PMC13427082; doi:10.1590/0074-02760260088)
Supplement: Peer review history

# Reviewer report: FIRST REVIEW ROUND - REVIEWERS' COMMENTS

## REVIEWER #1

The manuscript “Chikungunya Vaccine Approval in Brazil: Navigating Through Epidemiological Challenges and Immunization Strategies” analyzes chikungunya notification data from 2016 to 2023 and investigates correlations with demographic and socioeconomic variables (IDHM, including its income, education, and longevity components) across 5,570 Brazilian municipalities. The authors identify correlations between CHIKV notification rates and socioeconomic indicators and stratify municipalities into high-, middle-, and low-SES categories, along with their respective notification rates. Based on these findings, they propose a hybrid immunization strategy that prioritizes vaccination in low-SES municipalities to prevent outbreaks in high-SES areas, while reserving vaccination in high-SES municipalities for imminent outbreak scenarios.

Major Critique

The association between socioeconomic status (SES) and arboviral disease notification is well established, and heterogeneity in incidence is frequently observed within municipalities. While municipality-level stratification may offer operational convenience for immunization programs, it oversimplifies local epidemiological realities.

Although the correlation analysis with SES is adequately presented, the manuscript does not provide sufficient analytical support for the proposed immunization strategy. The claim that this approach is superior to alternative strategies is not substantiated. Critical factors such as population density, environmental variability, and proxy indicators associated with vector abundance—particularly across Brazil’s diverse geographic regions—are not adequately considered. For example, water shortages in São Paulo have previously triggered large outbreaks, underscoring the importance of contextual environmental drivers.

The discussion is overly speculative and excessively long. Several interpretations attempting to explain regional differences in CHIKV epidemiology are not consistent with established patterns observed in other arboviral diseases and are contradicted by recent events, such as the 2024 dengue epidemic.

Ethical considerations are insufficiently addressed. Public acceptance of vaccination is increasingly influenced by individual risk–benefit perceptions, particularly regarding potential adverse effects. The concept of collective benefit is progressively being deprioritized by the population. Addressing this shift requires transparency, including clear communication of both individual and community-level risks, as well as the frequency and severity of adverse events. The manuscript does not adequately discuss vaccine safety profiles, including the incidence and types of side effects or age-related differences in vaccine response. Notably, IXCHIQ has been associated with significant adverse effects in older populations, raising concerns about the ethical implications of proposing broad vaccination strategies targeting lower-SES populations primarily to protect higher-SES groups.

Minor Critique

The use of “notification rate” as a proxy for incidence may be practical but is inherently limited. There is insufficient evidence to establish how notification data correlate with true infection rates or seroprevalence across municipalities. Reporting efficiency likely varies substantially, with some municipalities demonstrating more robust surveillance systems than others. Therefore, it is recommended that notification rates not be equated with incidence.

## REVIEWER #2

The abstract is adequate. This contribution is relevant and original. Methodology is adequate. Results, Discussion, References and figures and table are appropiate.

Comments:

* Pages 1 and 5 (lines 55/56): Use the same pattern to 1st letters.

* Pages 5 (lines 9/10): Adjustment needed: CHIKV causes also chronic disease. Maybe changing for “can cause”, or adding “usually”, will be enough.

* Page 6 (line 10): Same previous observation.

* Page 7 (line 8): “hyperendemic country for CHIKV” sounds better.

* Page 7 (line 26): “conforming” sounds better.

* Page 7 (line 41): confirmed or suspected cases? Please inform these along the whole text, including the table and the figures.

* Page 11 (lines 43/44): I guess it is necessary to be cited in the analysis that CHIKD is underdiagnosed in Brazil and the difficulty in diagnosing may also vary in different regions, reflecting regional Health assistance disparities.

* Page 14 (lines 13/14): It may be useful to discuss that overlapping arboviruses (dengue, zika, oropouche, west nile...) in the same areas, but with different prognosis and managements. This facts highlights the need for vaccine strategy.

* Page 15 (line 38): As a suggestion, to be considered by the authors, it should be pointed out that Ixchiq® is not now being used in some populations that would need priority for being more susceptible to severity (elderly, neonates, those with underlying conditions...) due to the nowadays abscence of proven efficacy/safety, or to real life related side effects.

Page 20 (line 11): Adjust the “r” in cluster (table).

## AUTHORS' RESPONSE TO THE REVIEWERS

Response to the reviewer comments/ criticisms

Reviewer: 1

*The manuscript “Chikungunya Vaccine Approval in Brazil: Navigating Through Epidemiological Challenges and Immunization Strategies” analyzes chikungunya notification data from 2016 to 2023 and investigates correlations with demographic and socioeconomic variables (IDHM, including its income, education, and longevity components) across 5,570 Brazilian municipalities. The authors identify correlations between CHIKV notification rates and socioeconomic indicators and stratify municipalities into high-, middle-, and low-SES categories, along with their respective notification rates. Based on these findings, they propose a hybrid immunization strategy that prioritizes vaccination in low-SES municipalities to prevent outbreaks in high-SES areas, while reserving vaccination in high-SES municipalities for imminent outbreak scenarios.*

Reply: We are very grateful to the reviewer for the careful reading of the presented work and providing this summary. We would like to clarify that the proposed hybrid immunization strategy is presented as a conceptual framework to guide prioritization under multiple circulation of different arboviruses, supposed vaccine limitation and strong dissemination of CHIKV rather than a vaccine policy recommendation strategy. In the revised version of the manuscript, we further contextualize this point along with other criticisms raised by the reviewer.

Thank you very much for the suggestions.

Major critique

*The association between socioeconomic status (SES) and arboviral disease notification is well established, and heterogeneity in incidence is frequently observed within municipalities. While municipality-level stratification may offer operational convenience for immunization programs, it oversimplifies local epidemiological realities.*

Reply: We thank the reviewer for this important observation. We agree that the heterogeneity of the arboviral diseases frequently can occur within municipalities and that municipality level analyses may simplify complex local arboviral epidemiology.

We, however, emphasize, that the municipality represents the primary administrative and operational unit in Brazil for surveillance and implementation of vaccination strategies. Therefore, epidemiological analyses at this level are particularly relevant for public health decision-making and consequently resource allocation. Our intention was not to imply homogeneity within municipalities but rather to identify broader spatial patterns that can support prioritization under real-world programmatic constraints, for example, limited vaccine availability.

Accordingly, we have revised our manuscript to explicitly acknowledge the ecological nature of the analysis and its limitations and accentuate that finer scale heterogeneity should be considered in vaccine implementation. The following modifications were included in the revised version of the manuscript:

Lines 230-234, Page 10: “...While we acknowledge that municipality-level data can mask intra-municipal heterogeneities of arbovirus circulation and this can simplify the complex arbovirus epidemiology. However, our findings corroborate with previous studies ^13^ and also emphasize that the municipality is the primary administrative and operational unit in Brazil for public health management and implementation of vaccination strategies…”

*Although the correlation analysis with SES is adequately presented, the manuscript does not provide sufficient analytical support for the proposed immunization strategy. The claim that this approach is superior to alternative strategies is not substantiated. Critical factors such as population density, environmental variability, and proxy indicators associated with vector abundance—particularly across Brazil’s diverse geographic regions—are not adequately considered.*

Reply: We are grateful to the reviewer for this thorough and constructive comment. We completely agree that the performed analysis does not constitute a formal comparative evaluation of immunization strategies and the original wording may have overstated the evidence supporting the proposed approach.

In response of this, we have revised our manuscript clarifying that the proposed hybrid immunization strategy should be interpreted as hypothesis generating and conceptually informed framework, rather than a primary and superior to other immunization strategies. We have removed language stating such ideas from the “Discussion” section and stress the use under factors of potential restrain of vaccine doses.

We completely acknowledge that important factors such as population density, environmental stability, and proxy indicators were not incorporated in the present analysis. These variables, are thus indeed important for issues related to transmission dynamics and their inclusion is beyond the scope of this study which focused on nationally available and standardized indicators.

We have addressed this as a limitation in the “Discussion” section to explicitly recognized these unmeasured factors and the need of further studies integrating epidemiological, environmental and entomological data in more granular and spatially explicit models to better inform vaccination strategies in Brazil´s heterogenous settings. The following information was added to the “Discussion” section:

Lines 238-244, Page 10: “... While our study focuses exclusively on standardized socioeconomic indicators, we acknowledge that the absence of correlation of environmental and entomological variables, such as vector density and water scarcity can be regarded as a limitation of our analysis. These factors, however, are important for investigations of the transmission dynamics of CHIKV and their inclusion is beyond the scope of this study which is focused is focused on nationally available and standardized indicators…”

*For example, water shortages in São Paulo have previously triggered large outbreaks, underscoring the importance of contextual environmental drivers.*

Reply: We are grateful to the reviewer for highlighting the importance of environmental and contextual drivers in shaping arbovirus dynamics. We agree that factors such as climate variability, water availability, and human behavior responses can influence vector proliferation and incidence of arboviral diseases.

While specific situations such as water scarcity may contribute to increased transmission through water storage practices (mosquito egg deposits), we note that in Brazil, chikungunya, along with other arboviral outbreaks including those caused by dengue and zika viruses are more consistently associated with seasonal rainfall patterns which directly affect the availability of breeding sites of mosquitos of the *Aedes* genus.

To address this point, we have revised the manuscript to acknowledge the role of environmental and ecological determinants including, but not limited to, rainfall patterns, temperature and urban infrastructure-while avoiding oversimplified or context-specific patterns. As such patterns were not a direct objective of this study, they were not explicitly modelled in the revised version of the manuscript but in the future they should be incorporated into more detailed studies.

We have significantly refined the Discussion section to be more concise and have moderated our claims regarding the immunization strategy. We now present it as a precision public health framework rather than a standalone superior alternative. However, we maintain that our statistically significant spatial cluster analysis provides robust analytical evidence that CHIKV transmission is non-uniform and highly concentrated in socio-environmental niches. By identifying these non-random hotspots, our study demonstrates that SES acts as a powerful integrative proxy for vulnerability, offering a more targeted approach than traditional, aggregate municipality-level data. While we have now included the absence of direct environmental and entomological variables (e.g., water scarcity and vector density) as a study limitation, these factors do not invalidate the identified spatial groupings, which remain critical for optimizing resources in high-burden areas.

We added the following information in the discussion section:

Lines 235-238, Page 10: “… By delineating territorial patterns of vulnerability at the primary administrative level used by the Brazilian Unified Health System (SUS), this analytical technique guides the more effective allocation of resources, the targeting of epidemiological surveillance actions, and the formulation of focused public policies, especially in historically neglected regions.^14^…”

Line 200, Page 8 and Lines 201-207, Page 9: “... Crucially, our analysis accounts for the fact that CHIKV is significantly underdiagnosed in Brazil, with diagnostic challenges and health assistance disparities varying greatly across regions. We acknowledge that notification rates serve as a proxy for disease burden rather than true infection incidence, as reporting efficiency is inherently influenced by regional variations in surveillance capacity. Given the rapid CHIKV expansion from the Northeast to the Southeast /South regions, the approval of the CHIKV vaccine by the Brazilian Regulatory Health Agency (ANVISA) in 2025 is expected to gain rapid incorporation in the Brazilian Public Health Services….”

*The discussion is overly speculative and excessively long. Several interpretations attempting to explain regional differences in CHIKV epidemiology are not consistent with established patterns observed in other arboviral diseases and are contradicted by recent events, such as the 2024 dengue epidemic.*

Reply: We are grateful for the reviewer for this critical observation. We completely agree that the “Discussion” section in its original form was extensive and in some parts speculative. In response to the reviewer comment, we have substantially revised the Discussion section to improve focus and clarity. We have given an additional focus on empirical data reducing speculative interpretations.

However, we would like to clarify respectfully that the epidemiological dynamics of CHIKV and DENV are distinct, despite sharing the similar mosquito vector. Based on the above shown literature support, we consider that CHIKV patterns observed in our study may diverge from the 2024 dengue epidemic:

Biological and Thermal Thresholds: As demonstrated by Mordecai et al. (2017, PLOS NTDs), CHIKV has specific thermal requirements and transmission thresholds that differ from DENV. Their mechanistic models show that climate-driven responses are not uniform across arboviruses, explaining why CHIKV geographical expansion may not mirror DENV.

Epidemiological Interactions and Immunity: Evidence from long-term time series (e.g., Ecuador Study, 1988–2024) reinforces that CHIKV, DENV, and ZIKV exhibit virus-specific interaction patterns and climate drivers. The 2024 dengue surge is largely driven by multi-serotype dynamics and population susceptibility. In contrast, CHIKV is constrained by long-term, sterilizing immunity from a single serotype, which fundamentally alters its regional recurrence patterns compared to dengue.

We added the following modification in the “Discussion” section:

Lines 219_225, Page 9: “… It is essential to distinguish CHIKV dynamics from other arboviruses like DENV. Unlike the multi-serotype complexity of DENV, CHIKV transmission is fundamentally constrained by long-term immunity to a single infecting serotype. Furthermore, CHIKV has specific thermal thresholds and transmission models that do not necessarily mirror the patterns observed in the 2024 Dengue epidemic, explaining its unique regional recurrence patterns with implications for economic stability, healthcare system, and challenges for vector control, underscoring the importance of adaptive public health policies, particularly in urban environments^12^.

*Ethical considerations are insufficiently addressed. Public acceptance of vaccination is increasingly influenced by individual risk–benefit perceptions, particularly regarding potential adverse effects. The concept of collective benefit is progressively being deprioritized by the population. Addressing this shift requires transparency, including clear communication of both individual and community-level risks, as well as the frequency and severity of adverse events.*

Reply: We are grateful to the reviewer for raising this important point. We agree with the reviewer that ethical considerations and public perception of risk are paramount for the success of any immunization strategy. We have updated the Discussion to address the shifting paradigm from collective to individual risk-benefit perception.

We now argue that our cluster-based approach actually supports ethical transparency: by identifying high-incidence ‘hotspots,’ public health authorities can provide local populations with precise information about their specific infection risk. This clear communication of community-level risk helps individuals make more informed decisions, balancing the potential for adverse events against the high probability of infection in their immediate environment. We have also emphasized the need for transparent reporting of adverse event frequencies to maintain public trust.

The following information has been added to the revised version of the manuscript:

Lines 283-290, Page 12: “... The ethical imperative of CHIKV immunization in Brazil lies in integrating epidemiological evidence with principles of social justice, while navigating the shifting paradigm of public vaccine acceptance. As individual risk–benefit perceptions increasingly outweigh traditional collective benefits, addressing this shift requires radical transparency in risk communication. By identifying high-burden ‘hotspots,’ our framework allows health authorities to provide precise, community-level data, enabling individuals to make informed decisions by weighing their localized infection risk against the vaccine’s safety profile. …”

*The manuscript does not adequately discuss vaccine safety profiles, including the incidence and types of side effects or age-related differences in vaccine response. Notably, IXCHIQ has been associated with significant adverse effects in older populations, raising concerns about the ethical implications of proposing broad vaccination strategies targeting lower-SES populations primarily to protect higher-SES groups.*

Reply: We appreciate the reviewer’s critical ethical reflection. We agree that vaccine safety profiles including incidence and spectrum of adverse effects and potential age-related differences are critical considerations for any immunization strategies. We have revised the “Discussion” section to include a detailed analysis of the vaccine’s safety profile, specifically addressing the adverse events associated with the IXCHIQ vaccine, including evidence for its effects in different age groups. We emphasize that vaccination strategies should be aligned with regulatory guidance and contraindications particularly in populations that might be at higher risk for adverse reactions outcomes. We have also explicitly addressed the ethical concern regarding vaccination in lower-SES populations. We clarify that our cluster-based strategy is designed to prioritize individuals at the highest risk of infection — who, in our study, are concentrated in lower-SES areas — to reduce their own disease burden and potential for chronic sequelae. We have removed any phrasing that could imply a utilitarian use of one population to protect another, emphasizing instead the principle of equity: providing interventions where the risk of disease is objectively higher. Therefore, our study aims to identify areas where vaccination could have greatest impact on transmission dynamics under conditions of vaccine limitation while maintaining equity and adherence to safety considerations.

Additionally, we now discuss the need for age-stratified safety monitoring, particularly for older populations in these clusters, to ensure that the risk-benefit ratio remains favorable.

The following modifications were added to the manuscript text:

Lines 269-274, Page 11 and Lines 275-278, Page 12: “… Furthermore, the current clinical limitations of available vaccines must be addressed; for instance, Ixchiq® is a live-attenuated vaccine and its reactogenicity profile requires careful assessment, particularly in pediatric patients, immunosuppressed individuals and older populations who may face a higher frequency of systemic adverse events. Currently this vaccine, it is not indicated for certain high-risk groups, such as neonates, pregnant women, and severely immunocompromised individuals, due to the present lack of comprehensive safety and efficacy data for these specific populations. This restriction poses a significant challenge, as these groups, along with the elderly, who may be more susceptible to vaccine-related adverse events are often the most susceptible to severe CHIKV complications. …”

Minor critique

*The use of “notification rate” as a proxy for incidence may be practical but is inherently limited. There is insufficient evidence to establish how notification data correlate with true infection rates or seroprevalence across municipalities. Reporting efficiency likely varies substantially, with some municipalities demonstrating more robust surveillance systems than others. Therefore, it is recommended that notification rates not be equated with incidence.*

Reply: We thank the reviewer for this important methodological observation. We agree that notification rates are imperfect proxy for true incidence and may be influenced by variations in surveillance capacity, reporting practices and healthcare access across municipalities.

We would like to clarify that our intention was not to equate notification rates with true infection incidence but rather to use them as standardized and nationally available indicator to explore spatial and socioeconomic patterns of CHIKV reported cases.

In response to this comment, we have revised the manuscript text to consistently refer to “notification rates” rather than “incidence” where appropriate and to explicitly acknowledge the limitations associated with underreporting and heterogenous surveillance systems. Despite these limitations, notification data remain a key component for public health surveillance in Brazil and provide valuable insights for comparative and programmatic analyses, particularly in the absence of uniformly available seroprevalence data.

The following modification was performed in the “Methods” section:

Lines 103-105, Page 4: “... In this study, notification rates are used as a proxy for disease burden, acknowledging that these figures may not reflect true incidence due to inherent underreporting and regional variations in surveillance systems…”

Reviewer: 2

The abstract is adequate. This contribution is relevant and original. Methodology is adequate. Results, Discussion, References and figures and table are appropriate.

We are grateful to the reviewer for the positive evaluation of the manuscript and for recognizing its relevance and originality. We appreciate the assessment that the overall presentation of the manuscript.

We have carefully considered all comments provided and have revised the manuscript in accordance with your suggestions. Thank you very much!

Comments:

* Pages 1 and 5 (lines 55/56): Use the same pattern to 1st letters.

Reply: Changes performed.

* Pages 5 (lines 9/10): Adjustment needed: CHIKV causes also chronic disease. Maybe changing for “can cause”, or adding “usually”, will be enough.

Reply: We are grateful to the reviewer for the valuable suggestion. We agree that CHIKV infection can also lead to chronic manifestations.

In response, we have revised the text, incorporating the correct wording for the conditions caused by CHIKV (please refer to Line 35, Page 2 and Lines 61-62, Page 3).

* Page 6 (line 10): Same previous observation.

Reply: We have performed modifications in the manuscript text.

* Page 7 (line 8): “hyperendemic country for CHIKV” sounds better.

Reply: We have performed this modification in the “Discussion” section (Line 196, Page 8).

* Page 7 (line 26): “conforming” sounds better.

Reply: We have performed modifications in the manuscript text (Line 94, Page 4).

* Page 7 (line 41): confirmed or suspected cases? Please inform these along the whole text, including the table and the figures.

Reply: We are grateful to the reviewer for this important methodological consideration. We agree that the distinction between confirmed and suspected cases is essential for the accurate interpretation of surveillance data. In response, we affirm the use of combined notification data. The following modifications were performed in the manuscript text:

Lines 100-101, Page 4: “...on notified suspected and confirmed CHIKV cases …”

* Page 11 (lines 43/44): I guess it is necessary to be cited in the analysis that CHIKD is underdiagnosed in Brazil and the difficulty in diagnosing may also vary in different regions, reflecting regional Health assistance disparities.

Reply: We are grateful to the reviewer for this important comment. We agree, that in many Brazilian regions, CHIKV infection may be underdiagnosed and that diagnostic capacity may depend on the healthcare services which may vary across different Brazilian regions, potentially reflecting underlying disparities in healthcare infrastructure and surveillance performance.

In order to reflect this point, we have revised the manuscript accordingly and have included discussion reflecting this and the appropriate references supporting underdiagnosis and regional differences in the detection of CHIKV. We also discuss how these factors may influence notification data and contribute to the observed spatial differences in the observed CHIKV cases. The following modifications were performed in the manuscript text:

We have added the following modification in the manuscript text:

Line 200, Page 8 and Lines 201-207, Page 9: “...Crucially, our analysis accounts for the fact that CHIKV is significantly underdiagnosed in Brazil, with diagnostic challenges and health assistance disparities varying greatly across regions. We acknowledge that notification rates serve as a proxy for disease burden rather than true infection incidence, as reporting efficiency is inherently influenced by regional variations in surveillance capacity. Given the rapid CHIKV expansion from the Northeast to the Southeast /South regions, the approval of the CHIKV vaccine by the Brazilian Regulatory Health Agency (ANVISA) in 2025 is expected to gain rapid incorporation in the Brazilian Public Health Services. …”

* Page 14 (lines 13/14): It may be useful to discuss that overlapping arboviruses (dengue, zika, oropouche, west nile...) in the same areas, but with different prognosis and managements. This facts highlights the need for vaccine strategy.

Reply: We are grateful to the reviewer for this important suggestion.

We acknowledge that the circulation of overlapping arboviruses in Brazil is common, including dengue, zika, yellow fever and most recently oropouche in the same areas and this a critical public health problem in Brazil.

Although these viral agents can show overlapping clinical symptoms they may differ in prognosis and clinical management and this additionally may complicate diagnosis when this is only based on clinical symptoms and lead to underrecognition or misclassification of cases. This epidemiological context is extremely important for robust and targeted vaccination strategies for vaccine preventable diseases, such as CHIKV.

For that reason, we performed modifications in the “Discussion” section highlighting arbovirus co-circulation and its implication for surveillance, clinical management and strategic value of vaccination programs in endemic settings. We have added the following information in the discussion section:

Lines 246-249, Page 10 and Lines 250-253, Page 11: “...and the complex clinical landscape characterized by the co-circulation of multiple arboviruses. In many Brazilian regions, CHIKV circulation overlaps with other important arboviruses like DENV, ZIKV and Oropouche . Since these infections often present with similar symptomatology but require distinct clinical management and have different prognoses, the risk of misdiagnosis is high. Such a diagnostic and clinical challenge often present with arbovirus diseases that further underscores the strategic importance of targeted CHIKV vaccine in Brazil, to reduce its burden and simplify the differential diagnosis in clinical settings. …”

* Page 15 (line 38): As a suggestion, to be considered by the authors, it should be pointed out that Ixchiq® is not now being used in some populations that would need priority for being more susceptible to severity (elderly, neonates, those with underlying conditions...) due to the nowadays abscence of proven efficacy/safety, or to real life related side effects.

Reply: We are grateful for the valuable comment of the reviewer.

We agree that current evidence regarding efficacy and safety in high-risk populations especially elderly, neonates and patients with underlying conditions, or immune suppression is still evolving and post-marketing surveillance and real-world data are essential to better define risk-benefit profile of this vaccine the above referred groups.

We understand, that this point can be discussed as a limitation of the vaccine and therefore we increment this line in the “Discussion” section including uncertainties regarding safety effectiveness and application in high-risk groups. We added the following information in the manuscript text:

Lines 269-274, Page 10 and Lines 250-253, Page 11: “… Furthermore, the current clinical limitations of available vaccines must be addressed; for instance, Ixchiq^®^ is a live-attenuated vaccine and its reactogenicity profile requires careful assessment, particularly in pediatric patients, immunosuppressed individuals and older populations who may face a higher frequency of systemic adverse events. Currently this vaccine, it is not indicated for certain high-risk groups, such as neonates, pregnant women, and severely immunocompromised individuals, due to the present lack of comprehensive safety and efficacy data for these specific populations. This restriction poses a significant challenge, as these groups, along with the elderly, who may be more susceptible to vaccine-related adverse events are often the most susceptible to severe CHIKV complications. ….”

*Page 20 (line 11): Adjust the “r” in cluster (table).*

Reply: We have modified the table in the revised version of the manuscript. Thank you for the observation.

# Reviewer report: REVIEWERS' COMMENTS

## REVIEWER #1

There are two minor comments:

1) IXCHIQ had an accelerated approval with several conditions to be meet. However subsequent analyzes of the side effects the approval was suspended and later the application was withdrawn by Vaneva. So, IXCHIQ does not have approval in the USA.

2) The clinical management of arbovirosis in Brazil is determined by clinical parameters not by the definition of the virus, dengue, Zika or Chikungunya. Often, the confirmatory diagnostic is not performed and seldom rapid tests are used at the point of care. So, I suggest refrain from making comments related to clinical management. The article does not have anything to do with this. If there was an effective drug than the diagnostic definition would be more relevant.

## REVIEWER #2

The new manuscript version responds to all previous observations made and requires minor adjustments to be published;

- Page 15, line 42: “confirmed” may be changed to “notified” to be in accordance to adjustments in methods.section;

- Page 16, line 62: the change made did not resolve the questions previouly pointed out. So, I suggest it would be better write “can cause an acute...”;

- Page 16, line 82: remove space between 2023 and 2024.

- Page 19, line 152: remove final “s” in patterns;

- Page 22, line 205: remove space between “Southeast South”;

- Page 23, line 230: “While” may be suppressed or the sentence rewritten;

- Page 23, line 240: association is more appropriate than “correlation;

- Page 23, line 243: “is focused” is repeated;

- Page 24, line 254: remove “have”;

- Page 24, line 273: this comma should be replaced right after “Currently”.

## AUTHORS' RESPONSE TO THE REVIEWERS

Reply to the reviewer comments/criticisms

Reviewer: 1

*There are two minor comments:*

*1) IXCHIQ had an accelerated approval with several conditions to be meet. However subsequent analyzes of the side effects the approval was suspended and later the application was withdrawn by Vaneva. So, IXCHIQ does not have approval in the USA.*

We agree with the reviewer. We removed the United States from the list of countries where IXCHIQ® is approved for use.

*2) The clinical management of arbovirosis in Brazil is determined by clinical parameters not by the definition of the virus, dengue, Zika or Chikungunya. Often, the confirmatory diagnostic is not performed and seldom rapid tests are used at the point of care. So, I suggest refrain from making comments related to clinical management. The article does not have anything to do with this. If there was an effective drug than the diagnostic definition would be more relevant.*

We agree with the reviewer. We modified this part in the text removing the clinical management from the “Discussion” section.

Reviewer: 2

*The new manuscript version responds to all previous observations made and requires minor adjustments to be published;*

We are grateful to the positive feedback of the reviewer regarding the presented revised version. Thank you very much.

*- Page 15, line 42: “confirmed” may be changed to “notified” to be in accordance to adjustments in methods.section;*

This change was performed.

*- Page 16, line 62: the change made did not resolve the questions previouly pointed out. So, I suggest it would be better write “can cause an acute...”;*

This change was performed

*- Page 16, line 82: remove space between 2023 and 2024.*

This space was removed.

*- Page 19, line 152: remove final “s” in patterns;*

“S” was removed from patterns.

*- Page 22, line 205: remove space between “Southeast South”;*

This space was removed.

*- Page 23, line 230: “While” may be suppressed or the sentence rewritten;*

“While” was removed from the sentence.

*- Page 23, line 240: association is more appropriate than “correlation;*

We changed “correlation” with “association”.

*- Page 23, line 243: “is focused” is repeated;*

We removed the repeated “is focused”.

*- Page 24, line 254: remove “have”;*

We removed “have” from the sentence.

*- Page 24, line 273: this comma should be replaced right after “Currently”.*

We placed the comma after “currently”.

**AUTHORS’ RESPONSE TO THE REVIEWERS**

Reply to the reviewer comments/criticisms

Reviewer: 1

*There are two minor comments:*

*1) IXCHIQ had an accelerated approval with several conditions to be meet. However subsequent analyzes of the side effects the approval was suspended and later the application was withdrawn by Vaneva. So, IXCHIQ does not have approval in the USA.*

We agree with the reviewer. We removed the United States from the list of countries where IXCHIQ® is approved for use.

*2) The clinical management of arbovirosis in Brazil is determined by clinical parameters not by the definition of the virus, dengue, Zika or Chikungunya. Often, the confirmatory diagnostic is not performed and seldom rapid tests are used at the point of care. So, I suggest refrain from making comments related to clinical management. The article does not have anything to do with this. If there was an effective drug than the diagnostic definition would be more relevant.*

We agree with the reviewer. We modified this part in the text removing the clinical management from the “Discussion” section.

Reviewer: 2

*The new manuscript version responds to all previous observations made and requires minor adjustments to be published;*

We are grateful to the positive feedback of the reviewer regarding the presented revised version. Thank you very much.

*- Page 15, line 42: “confirmed” may be changed to “notified” to be in accordance to adjustments in methods.section;*

This change was performed.

*- Page 16, line 62: the change made did not resolve the questions previouly pointed out. So, I suggest it would be better write “can cause an acute...”;*

This change was performed.

*- Page 16, line 82: remove space between 2023 and 2024.*

This space was removed.

*- Page 19, line 152: remove final “s” in patterns;*

“S” was removed from patterns.

*- Page 22, line 205: remove space between “Southeast South”;*

This space was removed.

*- Page 23, line 230: “While” may be suppressed or the sentence rewritten;*

“While” was removed from the sentence.

*- Page 23, line 240: association is more appropriate than “correlation;*

We changed “correlation” with “association”.

*- Page 23, line 243: “is focused” is repeated;*

We removed the repeated “is focused”.

*- Page 24, line 254: remove “have”;*

We removed “have” from the sentence.

*- Page 24, line 273: this comma should be replaced right after “Currently”.*

We placed the comma after “currently”.
